# High-intensity circuit training change serum myostatin but not myogenin in adolescents’ soccer players: a quasi-experimental study

**DOI:** 10.1186/s13102-023-00627-1

**Published:** 2023-02-06

**Authors:** Amirhosein Ziyaiyan, Mohammadreza Kordi, Martin Hofmeister, Karim Chamari, Wassim Moalla, Abbas Ali Gaeini

**Affiliations:** 1 Department of Sport Physiology, Faculty of Physical Education and Sports Sciences, University of Tehran, Tehran, Iran; 2 Department Food and Nutrition, Consumer Centre of the German Federal State of Bavaria, Munich, Germany; 3 Aspetar, Orthopedic and Sports Medicine Hospital, FIFA Medical Centre of Excellence, Doha, Qatar; 4 Laboratory EM2S LR19JS01: Education, Motricity, Sport and Health, High Institute of Sport and Physical Education of Sfax, University of Sfax, Sfax, Tunisia

**Keywords:** Interval training, Hypertrophy, Adaptation, Myogenin, Myostatin

## Abstract

**Background:**

Skeletal muscle contractions due to exercise lead to the secretion of many proteins and proteoglycan peptides called myokines. Myostatin (MSTN) and Myogenin (MyoG) are two of the most important skeletal muscle growth regulatory factors related to myoblast differentiation and muscle hypertrophy. The present study aims at investigating the effects over eight weeks of high-intensity circuit training (HICT) on serum MyoG and MSTN in male soccer players.

**Method:**

The present study is a quasi-experimental study on 21 male soccer players (Experimental group: n = 11, Control group: n = 10) (ages 15.0 ± 3.4 years, body mass 55.7 ± 7.8 kg, height 173.3 ± 8.0 cm, Body mass index 18.4 ± 1.9 kg m^−2^, maximum oxygen uptake 61.89 ± 3.01 ml kg^−1^ and the peak height velocity 14.5 ± 0.3 years). Participants were randomly divided into two groups: training group and a control group. The first resting blood samples were obtained in the morning-fasting state, and the second blood samples were obtained after the maximum aerobic test at pre- and post-HICT.

**Results:**

There were non-significant differences in resting serum values of MyoG (*p* = 0.309, *p* > 0.05) but significant differences in resting serum values of MSTN between the training and control groups after eight weeks of HICT (*p* = 0.003, *p* < 0.05). No significant differences were observed between groups in the acute response of serum values of MyoG (*p* = 0.413, *p* < 0.05) and MSTN (*p* = 0.465, *p* < 0.05) to the maximum aerobic test after eight weeks of HICT.

**Conclusion:**

These results suggest that eight weeks of HICT can decrease the resting serum values of MSTN but not change the resting serum values of MyoG in male adolescent soccer players. Also, eight weeks of HICT does not affect the acute response of MSTN and MyoG after a maximum aerobic test.

## Background

Nowadays, coaches are attempting to find a training protocol for adolescent athletes that, in addition to promoting physical and functional development in adolescents, also protects them from physiological stress and overtraining [1]. As muscle hypertrophy contributes to sports performance [2] and improves body composition [3], most athletes are looking for muscle hypertrophy.

Exercise could induce structural and metabolic changes in skeletal muscles [4]. These changes have been identified to cause skeletal muscle growth during various exercises, and adaptation could vary according to the exercise type through many signaling pathways [5]. High-intensity circuit training (HICT) by using body mass is one of the recommended practical and economical exercise methods since movements in circuit training are similar to daily activities (e.g., squat, climb stairs, etc.) and performed with minimum facilities [6]. Circuit training that combines multi-joint and Calisthenics training could keep the heart rate at high levels during a workout and increase physiological pressure due to alternate exercise and recovery (less than 15 s). Klika and Jordan [7] have also observed that the metabolic effects of interval training in adults remain up to 72 h after the training session. However, this type of training's metabolic effects on adolescents has not yet been thoroughly investigated [8].

In humans, skeletal muscle comprises about 40% of total body mass and 50–75% of body proteins [9]. Myokines are the cytokines and peptides secreted from skeletal muscle tissue with paracrine, endocrine, and autocrine effects [10]. Growth and hypertrophy are the primary physiological adaptations of skeletal muscle, which are affected by myogenic regulatory factors (MRFs) and transforming growth factor-beta (TGF-B) that play an essential role in the differentiation process of skeletal muscle cells by controlling transcription phenotype-specific proteins [11]. Among all the factors that regulate muscle growth, two factors, myogenin and myostatin regulate skeletal muscle growth in the postnatal period while strongly interacting with each other [12]. Myogenin (MyoG) is a myokine with a short life (half-life of 20–60 min) and is produced and released from muscles engaged in physical activity [13, 14]. MyoG induces muscle hypertrophy, satellite cell division, muscle fiber division, and other events and biological processes, such as hypertrophy and hyperplasia [15]. The process of skeletal muscle synthesis consists of three main steps, activation, proliferation, and differentiation [16]. Insulin-like growth factor 1 (IGF-1) is responsible for the growth process in humans, amongst others. Secretion of IGF-1 and mechano growth factor (MGF) during exercise can increase myogenin gene expression and production in skeletal muscle cells through the mammalian target of rapamycin complex 1 (mTOR c1) pathway [17]. External signals can make myoblasts active and proliferate and eventually increase MyoG and myoblast determination (MyoD) as critical factors for myoblast differentiation and fusion into myotubes [18]. Myostatin (MSTN) is an extracellular factor in the negative regulation of muscle mass with a half-life of approximately 2 h [19, 20]. MSTN, also known as growth/ differentiation factor-8 (GDF-8), is a GDF-beta family member [21]. Myostatin is a protein that binds to Activin receptor type IIB (ActRIIB) and disrupts the transcription of genes involved in myogenesis. Through MSTN, various pathways—TGF-β-activated kinase 1/Mitogen-activated protein kinase kinase (TAK-1/MAPKK) for p38 mitogen-activated protein kinase (p38 MAPKs) or rat sarcoma virus/rapidly accelerated fibrosarcoma/Mitogen-activated protein kinase kinase-1 (Ras/Raf/MEK1) for extracellular signal‐regulated kinases1/2 (ERK1/2)—activate MAPKs. It results in the suppression of myogenesis-related genes [22]. Importantly, Xiao et al. [21] have shown that inadequate recovery leads to anti-anabolic conditions and no change in MyoG values and myogenic factors. De Souza et al. [23] have also shown that excessive exercise intensity and inadequate recovery between training sessions can lead to muscle atrophy, an increase in MSTN and a reduction in MyoG.

Nowadays, many children and adolescents participate in sports all year long, often participating in several sports simultaneously. In this regard, overtraining may lead to burnout, harming a child's ability to participate in sports and physical activity as a lifetime healthy way of life [24]. Growth factors, especially around the growing peak at adolescence, are importance for maintaining athletes' physical and physiological growth while preventing developmental disorders caused by overtraining [24]. Obviously, this is also the case for soccer, which is very popular worldwide [25].

Therefore, considering that myogenin and myostatin are two important control factors in the final phase of muscle cell differentiation, they can be used as relevant indicators for evaluating training stress and recovery. Therefore, the purpose of this study was to investigate the effects of 8 weeks of HICT on serum values of MyoG and MSTN and on the acute response of serum values of MyoG and MSTN to a maximum aerobic test.

## Methods

### Participants

**Fig. 1:**
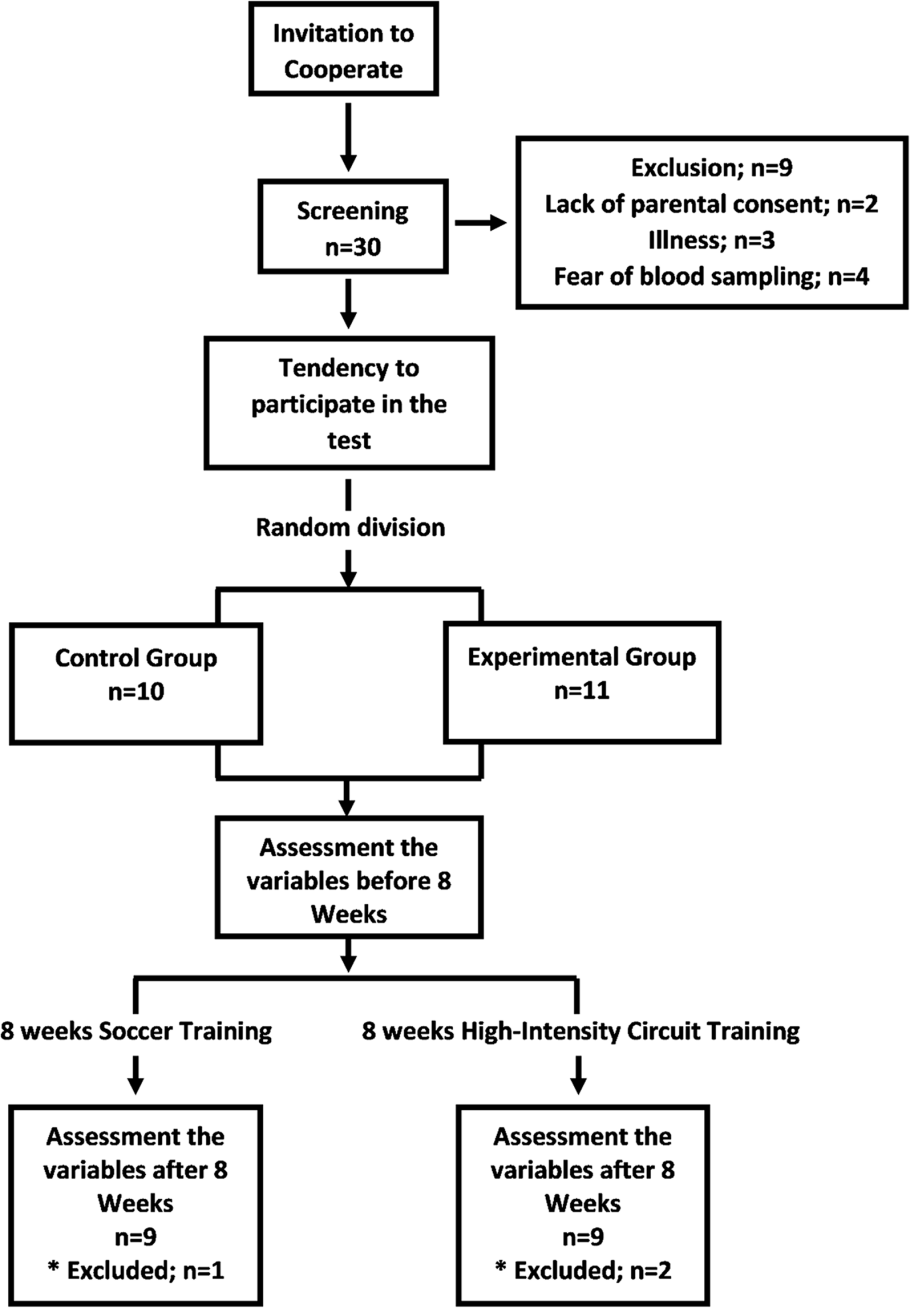
The flow chart of the entering process to research and the number of participants who completed the research process

Twenty-one male adolescent soccer players [age 15.0 ± 3.4 years, body mass 55.78 ± 7.82 kg, height 173.32 ± 8.01 cm, Body mass index (BMI) 18.49 ± 1.93 kg m^−2^, maximum oxygen uptake 61.89 ± 3.01 ml min^−1^ kg^−1^ and predicted age at peak height velocity (PHV) 14.54 ± 0.30 years] were volunteered to participate in this study. Eligible participants were also required to complete a Physical Activity Readiness Questionnaire (PAR-Q) to ensure a reasonably good health standing and physical preparedness, as well as demographic information by questionnaire and parental consent were obtained (Table 1, Fig. 1). The study was approved by the ethics committee of the University of Tehran and was conducted according to the standard of the declaration of Helsinky (Ethics Code: IR.UT.SPORT.REC.1398.015). Participants were selected by the available sampling method based on BMI and prediction of the age of PHV and divided randomly into two groups (HICT group; n = 11/control group; n = 10). Three individuals dropped out during the study, one due to illness (unrelated to the study), and two others did not attend all intervention sessions (Fig. 1). The characteristics of the participants are described in Table 1.

**Table 1:** Descriptive information about research participants. Training group; n = 9 and, Control group; n = 9

Variable	Training group (n = 9)		Control group (n = 9)	
	Mean	SD	Mean	SD
Age (year)	15.17	0.37	14.88	0.57
Weight (kg)	56.57	7.84	54.90	8.19
Height (cm)	174.05	9.53	172.50	6.38
BMI (kg m^−2^)	18.64	2.12	18.33	1.80
VO2max (ml kg^−1^ min^−1^)	63.11	2.85	60.53	2.70
PHV (year)	14.55	0.31	14.54	0.30

There was no significant difference between age, weight, height, body mass index (BMI), maximum oxygen consumption (VO2max) and prediction of age of peak height velocity (PHV) between groups (*p* > 0.05)

### Experimental design

**Fig. 2:**
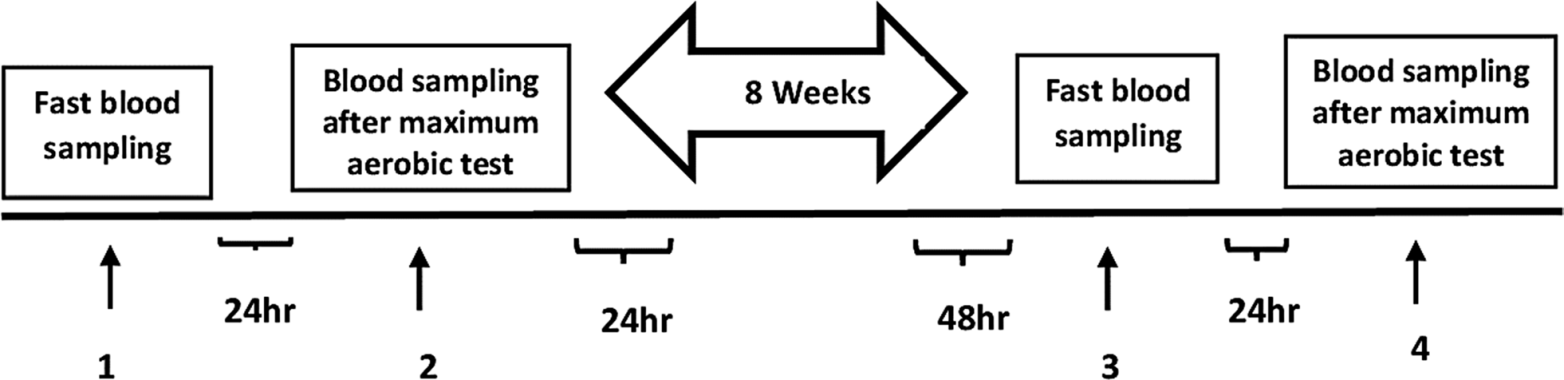
Schematic of the research design steps. Fasting blood samples were obtained after 2 days following the same diet by participants (Measuring Chronic response/Resting values of MyoG and MSTN). Twenty-four hours later, blood samples were obtained 1 h after maximum aerobic test (Measuring Acute response of MyoG and MSTN). Then, high-intensity circuit training (HICT) protocol was performed for 8 weeks. Fasting blood samples were obtained 48 h after the last training session (Measuring Chronic response/Resting values of MyoG and MSTN). Twenty-four hours later fasting blood samples, blood samples were obtained 1 h after the maximum aerobic test (Measuring Acute response of MyoG and MSTN)

In this quasi-experimental study, the training group performed the HICT protocol before starting of each soccer training session (HICT has been performed as part of the soccer training session in the training group while the control group was performing just soccer training. Therefore, the volume of training sessions was equal in both groups), three sessions per week for eight weeks (Fig. 2).

### HICT protocol

**Fig. 3:**
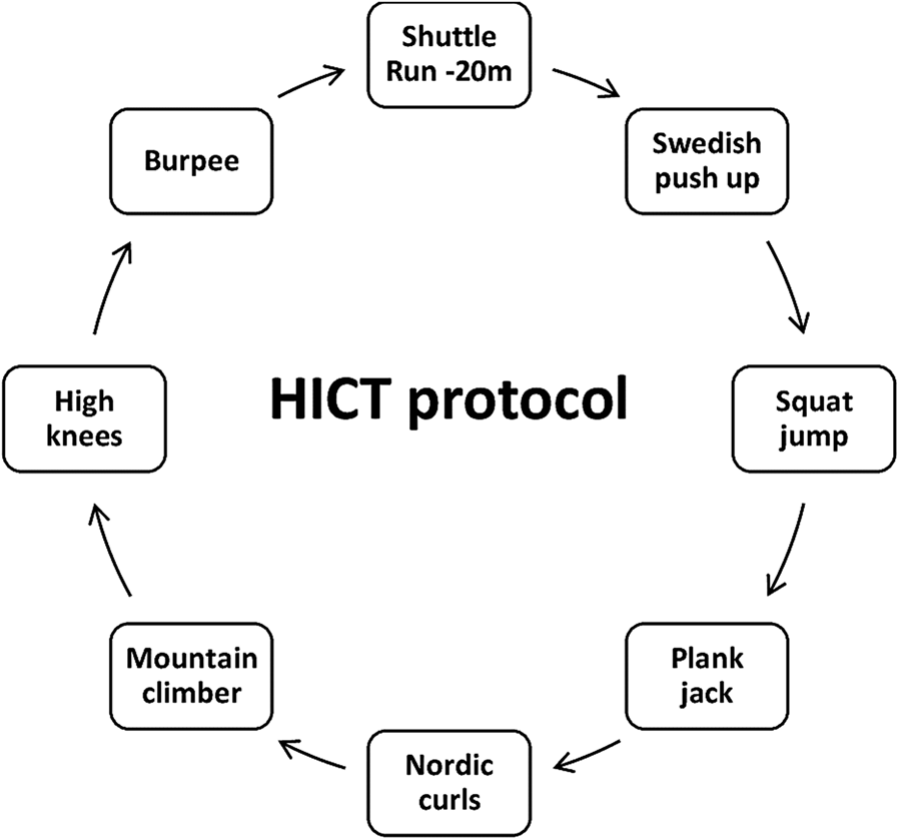
Exercise of each station in high-intensity circuit training protocol

The protocol consisted of eight stations: 20 m shuttle run, Swedish push-up, squat jump, plank jack, Nordic curls, mountain climber, high knees, and burpee (Fig. 3). These exercises were performed with body mass resistance. The Heart rate is checked by Polar heart rate monitor (FT7 model, made in Finland), and the rated perceived exertion is checked by Borg scale 20 units on a tablet. The protocol was performed in three sessions per week for eight weeks. In each session, participants performed the exercise of each station with maximum effort for 30 s (Borg scale more than 16 and heart rate greater than 80–85% maximum heart rate). At the end of the exercise, each participant had an active recovery by walking between stations. When the participant performed all stations once, it was considered one round. The training group performed one round (each station 14 min; 30 s activity, with 60 s resting between each station) on the first week, two rounds (each station 10 min, 30 s activity, with 45 s resting between each station), on the second week, three rounds (each station 8 min; 30 s activity, with 30 s resting between each station) on the third, fourth, fifth, sixth, seventh and eighth weeks (Table 2). The protocol was taken and adapted according to Klika and Jordan [7].

**Table 2:** High intensity circuit training (HICT) Protocol

Week	High intensity circuit training protocol				
	Weekly frequency	Intensity of each session	During each HICT (8 stations; 30 s)	Weekly volume	Volume of each session
First	3	Max exertion at 30” HR > 80% MHR	14 min Rest: 60”	3 HICT	1 HICT 14 min
Second	3	Max exertion at 30” HR > 80% MHR	10 min Rest: 45”	3 HICT	1 HICT 10 min
Third	3	Max exertion at 30” HR > 80% MHR	8 min Rest: 30”	6 HICT	2 HICT 8 min
Fourth	3	Max exertion at 30” HR > 85% MHR	8 min Rest: 30”	6 HICT	2 HICT 16 min
Fifth	3	Max exertion at 30” HR > 85% MHR	8 min Rest: 30”	9 HICT	3 HICT 24 min
Sixth	3	Max exertion at 30” HR > 85% MHR	8 min Rest: 30”	9 HICT	3 HICT 24 min
Seventh	3	Max exertion at 30” HR > 85% MHR	8 min Rest: 30”	12 HICT	4 HICT 32 min
Eight	3	Max exertion at 30” HR > 85% MHR	8 min Rest: 30”	12 HICT	4 HICT 32 min

After the start command, exercise of each station was performed with maximum effort for 30 s (Borg scale greater than 16 and heart rate (HR) more than 80–85% maximum heart rate (MHR); Participants walked as active resting between each station. When all participants performed all stations once, it was considered one round on the first week, two rounds (30 s activity, with 45 s resting between each station), on the second week, three rounds (each station; 30 s activity, with 30 s resting between each station) on the third, fourth, fifth, sixth, seventh and eighth weeks

### Running protocol

In this study, participants ran on a treadmill at a speed of 6 km h^−1^ for 3 min (warming up). The treadmill speed increased by 1 km h^−1^ for each minute. Participants continued running until exhaustion and reaching maximum heart rate with standard verbal encouragement provided [26]. Heart rate changes (reaching maximum heart rate by age-predicted) were checked by using a Polar heart rate monitor and, rating of perceived exertion (RPE) was obtained by the Borg scale (20 units) on a tablet during running. The test aimed to evaluate the effect of 8 weeks of HICT on the acute response of serum MyoG and MSTN to maximal aerobic activity (physiological conditions similar to soccer training/competition).

### Prediction of age of peak height velocity (APHV)

Based on growth patterns of legs and upper trunk, there is a calculation that, in up to 95% of cases, enables to predict the age of peak height velocity (APHV) in + 1 to − 1-year range. For calculating APHV, sitting height, leg length, body mass, and chronological age are required (Eq. 1). This method of maturity prediction is fast, non-invasive, and low-cost and could be used in cross-sectional studies such as the present study. The equation for calculating the Age of Peak Height Velocity is below [27].

1 $$\begin{aligned} {\text{Maturity}}\;{\text{Offset}} & = - 9236 + 0.0002708 \times {\text{leg}}\;{\text{length}}\;{\text{and}}\;{\text{sitting}}\;{\text{height}}\;{\text{interaction}} \\ & \quad {-}0.001663 \times {\text{Age}}\;{\text{and}}\;{\text{leg}}\;{\text{length}}\;{\text{interaction}} + 0.007216 \\ & \quad \times {\text{Age}}\;{\text{and}}\;{\text{sitting}}\;{\text{height}}\;{\text{interaction}} + 0.02292 \\ & \quad \times \;{\text{height}}\;{\text{by}}\;{\text{weight}}\;{\text{ratio}}\;[27] \\ \end{aligned}$$

### Biological measurements and analyses

All participants were instructed not to participate in any exercise (except soccer exercises) and consume any medications and sports supplements during the eight weeks of intervention. The blood sampling was done at four-time points in training and control groups. Fasting blood samples (Measuring the chronic response of MyoG and MSTN) were obtained from the cubital vein on the morning of the third day of assessment. Blood samples were obtained 1 h after the maximum aerobic test (Measuring the acute Response of MyoG and MSTN) to assess acute responses of MyoG and MSTN. All participants consumed the same snack meal after finishing the maximum aerobic test and blood sampling provided by the Laboratory expert team. The training group performed HICT three sessions per week for eight weeks. Forty-eight hours after the last training session, the fasting blood samples were obtained from the cubital vein. The next day, blood samples were obtained 1 h after the maximum aerobic test to assess the acute response of MyoG and MSTN (Fig. 2). After blood sampling, samples were placed at room temperature [20–22 °C (68–72 °F)] for 20 min for clotting, then the tubes were centrifuged for 15 min at 3000–3500 r min^−1^, and the serum was isolated in two separate microtubes. The samples were then immediately stored at − 80 °C for ~ 30 days. For measuring serum MyoG and MSTN, ELISA (EIA) Kits were used (Myogenin EIA kit: Bioassay Technology Laboratory, Cat. No E0421Hu, Shanghai, China/Myostatin EIA kit: Bioassay Technology Laboratory, Cat. No E0403Hu, Shanghai, China).

### Data analysis

Statistical analyses were conducted using a statistical software package (IBM SPSS Statistics 23.0, Armonk, NY: IBM Corp.). The Shapiro–Wilk and Levene’s test were used to verify the normality of distribution and homogeneity of variance, respectively. Analysis of Covariance was used to compare the mean values in the HICT and control groups. All data are presented as means ± standard deviation of the mean (SD). The significance values were set at *p* ≤ 0.05.

## Results

### Resting values (chronic response)

**Fig. 4:**
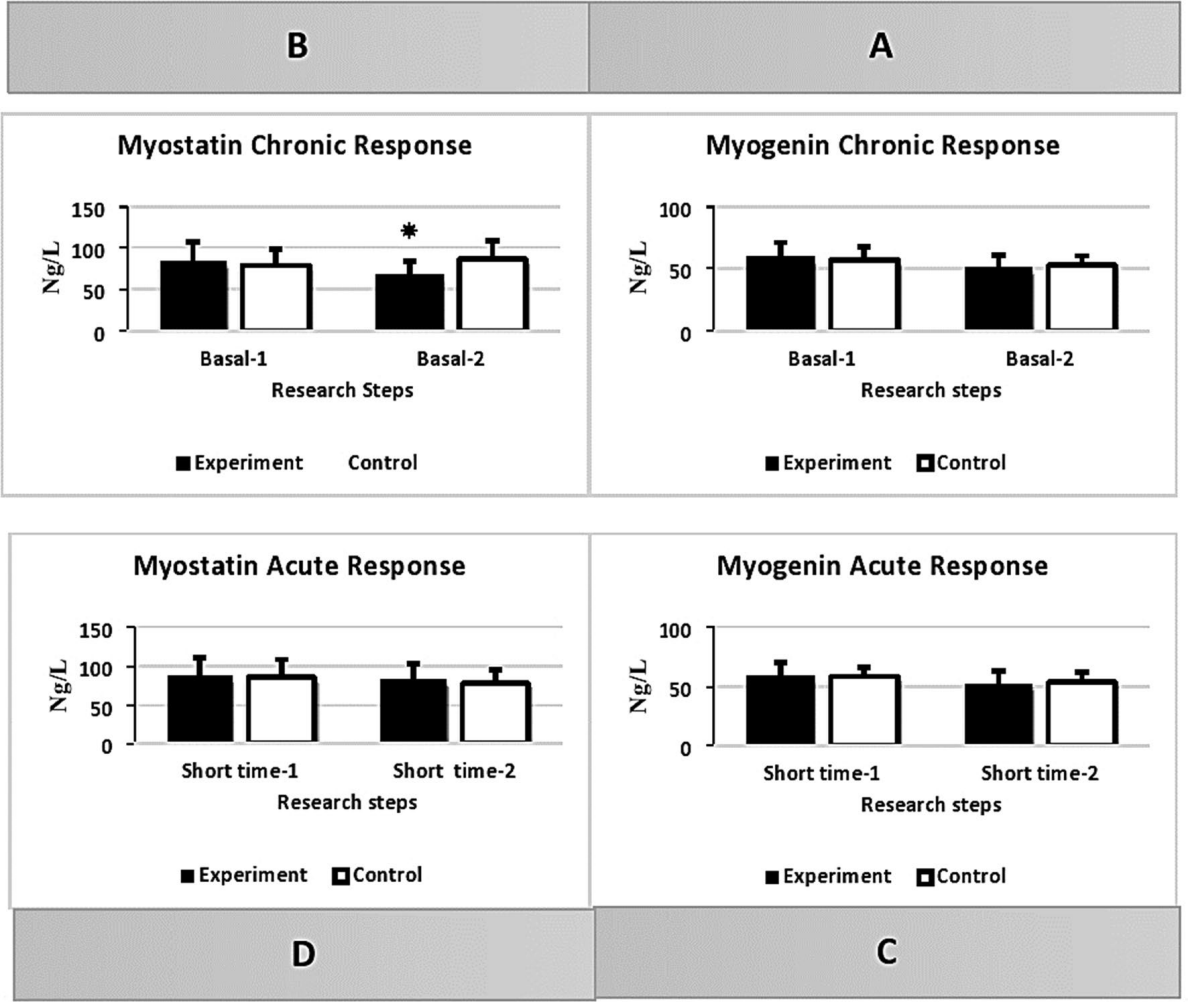
Comparison of intragroup and between groups indipendent variables. **A** Comparison of intragroup and between groups myogenin changes before and after the eight-weeks high-intensity circuit training (HICT). **B** Comparison of intragroup and between groups myostatin changes before and after the eight-weeks HICT. **C** Comparison of intragroup and between groups serum myogenin response to the maximum aerobic test, at pre and post eight weeks performing HICT. **D** Comparison of intragroup and between groups serum myostatin response to the maximum aerobic test, at pre and post eight weeks performing HICT

Compared to the pretest results, there was a non-significant difference in the mean resting values of MyoG serum between the training and control groups after eight weeks of HICT, whilst adjusting for soccer training [F(1,14) = 1.114, *p* = 0.309, η^2^ = 0.074]. But There was a significant difference in the mean resting values of MSTN serum between the training and control groups after eight weeks of HICT, whilst adjusting for soccer training [F(1,14) = 12.335, *p* = 0.003, η^2^ = 0.468]. The results showed resting values of MSTN serum decreased significantly in the training group compared to the control group (*p* ≤ 0.05, *p* = 0.003) (Table 3) (Fig. 4).

**Table 3:** The analyze of Covariance (ANCOVA) for comparison resting values of myogenin (MyoG) and myostatin (MSTN) serum after 8 weeks of high-intensity circuit training (HICT) and and acute response of MyoG and MSTN serum to the maximum aerobic test, after 8 weeks HICT between Training and Control groups, whilst adjusting for soccer training (ng/L)

Sorce	Mean*		*df*	F	Sig	Partial eta squared	Observed power
	Training	Control					
Group (Resting MyoG)	51.18	53.84	1	1.114	0.309	0.074	0.166
Group (Acute MyoG)	52.56	54.66	1	0.710	0.413	0.048	0.123
Group (Resting MSTN)	68.47	85.42	1	12.335	0.003	0.468	0.904
Group (Acute MSTN)	78.29	75.57	1	0.565	0.465	0.039	0.108

*Adjusted means (controlling for the covariate)

*Acute MyoG: Response of MyoG after the maximum aerobic test at pre- and post-HICT. Acute MSTN: Response of MyoG after the maximum aerobic test at pre- and post-HICT, Chronic MyoG: Response of MSTN during the rest time before and after eight weeks HICT

### Effect of adaptation on acute response (acute response to the maximum aerobic test)

The ELISA results showed a non-significant difference in the values of MyoG serum 1 h after the maximum aerobic between Training and Control groups after eight weeks of HICT, while adjusting for soccer training [F(1,14) = 0.710, *p* = 0.413, η^2^ = 0.048]. Likewise, there was a non-significant difference in the values of MSTN serum 1 h after the maximum aerobic test between Training and Control groups while adjusting for soccer training [F(1,14) = 0.565, *p* = 0.465, η^2^ = 0.039]. (Table 3) (Fig. 4).

## Discussion

The present study aimed to examine the effect of HICT protocol on muscle growth regulatory serum factors in adolescent male soccer players. The main findings of the present study showed that the resting serum values of MyoG in adolescent male soccer players did not change significantly after eight weeks of HICT, but the resting serum values of MSTN decreased significantly. Also, performing eight weeks of HICT did not significantly affect the acute response of serum MyoG and MSTN adolescent male soccer players to the maximum aerobic test.

By investigating the effect of adaptation on serum MyoG and MSTN’s acute response to the maximum aerobic test, no significant changes were observed in either training or control groups in comparison before and after eight weeks of HICT. Therefore, it can be concluded that eight weeks of HICT did not impact the acute response of serum MyoG and MSTN to the maximum aerobic test.

The current study showed that eight weeks of HICT did not show a difference in the resting serum values of MyoG in adolescent boy soccer players. In contrast to our data, Wilborn et al. investigated the effect of different intensities (60–65% 1RM and 80–85% 1RM) of resistance training on myogenic regulatory factors (MyoG, MRF4, Myf5, MyoD) in 13 young men recreationally active but nonresistance-trained [28]. Wilborn et al. showed a significant increase in the expression of myogenic regulatory factors in both training intensities [28]. This heterogeneity of the results is probably due to differences in variables (Protein and Gene). However, this study involved non-athlete; therefore, exercise could induce different molecular responses in trained and untrained human muscles [29]. Moreover, MRFs are transient regulatory proteins, like many transcription factors. The ubiquitin–proteasome system has been shown to degrade MyoD, Myf5, and myogenin [30]. Our results align with the findings of Costa et al., who showed no change in the expression values of myogenic factors on the seventh day after six days of repetitive bouts of eccentric exercise  [31]. Therefore, because Gene expression does not necessarily lead to protein translation [32], a change in MyoG gene expression does not necessarily lead to an increase in serum values of MyoG protein. Also another critical point that can be mentioned is recovery. Our participants were students, and due to their physical activity beyond gym exercises, their recovery may not have been adequate, which may have affected the results. Xiao et al. [21] and De Souza et al. [23] showed insufficient recovery between training sessions led to anti-anabolic conditions and reduction of MyoG, MyoD, and IGF-I. Also, Schwarz et al. [33] showed that lack of sleep reduced the cellular indicators of muscle repair in male rats. Yang et al. [34] found that lack of sleep reduced protein synthesis. In addition, in a recent review, Vlahoyiannis et al. [35] stated that young athletes, especially young athletes of Asian descent with low sleep quality. Thus, there are several reasons that may contribute to the change or absence of change in values of serum myogenin protein.

This study reported that eight weeks of HICT decreased the resting serum values of MSTN in boy adolescent soccer players significantly. Similarly to the present study, MSTN gene and protein expression decreased after 12 weeks of resistance training in young men [36]. A possible explanation for the similarity in findings may be the existence of resistance in high-intensity circuit training protocol. De Souza et al. [23], in a study, investigated the effects of concurrent strength and endurance training on genes associated with the MSTN signaling pathway and musculoskeletal responses. The results of this study showed that MSTN and its associated genes, activin, growth, and differentiation factor-associated serum protein-1 (GASP-1), forkhead transcription factor O subfamily member 3a (FOXo-3a), and IIB genes remained unchanged after eight weeks of combined training [23]. The reason for the contradictions among these studies is not apparent. These inconsistencies may be partly due to the differences in the protocol type, the intensity and duration of exercise, the gender and personal traits of the participants, the measurement method, and the timing for sampling. Moreover, we collected resting blood samples forty-eight hours after the last training session so that the acute effects of training had subsided. In that regard, it has been demonstrated that myostatin levels in the plasma of healthy young men dramatically drop 24 h after exercise compared to pre-exercise and that myostatin levels positively correlate with plasma Interleukin-6 (IL-6) [37]. The present study's findings were in accordance with those obtained by Bahram and Pourvaghar and Bagheri et al. [38, 39]. Bahram and Pourvaghar [38], about the effect of 10 weeks of resistance training on serum MSTN values in non-athlete obese adolescent boys, showed that resistance training for ten weeks significantly decreased plasma Myostatin values. Also, Bagheri et al. [39], after examining the effect of three categories, upper-trunk, lower-trunk, and combined lower and upper-trunk resistance training protocols for eight weeks on serum values of MSTN and follistatin in healthy middle-aged men, showed a significant decrease in serum values of MSTN in both lower-trunk and combined training protocols (upper trunk and lower trunk). These findings follow the current study. All these studies indicated that both regular and single-session resistance exercises reduced myostatin expression, thus promoting protein synthesis. This exercise-induced downregulation likely forms part of a beneficial hypertrophic adaptive response [40]. It is also worth mentioning that athletes of both sexes have a higher total antioxidant capacity than non-athletes, which in turn leads to better control and reduction of oxidants and inflammatory agents [41]. On the other hand, according to the relationship between inflammatory factors and serum myostatin levels, a further decrease in inflammatory factors may be associated with a further decrease in serum myostatin levels [42].

We are the first to show the preconditioning effect of HICT on the acute response of serum MyoG and MSTN to a maximum aerobic test. Most studies investigated the acute response of MyoG and MSTN to HICT and aerobic training. Our results showed non-significant changes in the acute serum values of MyoG to the maximum aerobic test after eight weeks of HICT in boy adolescent soccer players. The myokines investigated here play a critical role in muscle growth and metabolism; Although, on the one hand, Myf-5, MyoD, and myogenin gene transcription decrease in mature muscle tissue, MRF4 transcription, on the other hand, increases, making MRF4 the dominant MRF in adult muscle [43]. Coffey et al. showed that an acute cycling bout increased mRNA myostatin by twofold, MyoD by threefold, and MyoG by < onefold in endurance-trained subjects [44]. One study has reported increased myostatin expression after an acute resistive session [36]. In addition, an acute bout of resistance training can reduce [45] or not change [46] MyoG values and increase MyoD mRNA expression [44] in young but not elderly. Probably due to the catabolic condition of the body after training and to the increase of some pro-inflammatory factors due to the high intensity of training [47], which have inhibitory effects on the myogenic differentiation factors [48], in the present study, MyoG values remained unchanged. The effects of acute exercise on myostatin remain unclear, with some studies finding a downregulation of myostatin mRNA expression after different types of exercise [49–51] but others failing to find such changes [52, 53]. Although MacKenzie et al. [53] found a decrease in MSTN transcriptional activity after resistance exercise, the exercise stimulus activated Notch, a TGF-B inhibitor. The authors concluded that despite the acute increase in myostatin expression, inhibiting its transcriptional activity might contribute to exercise-induced skeletal muscle hypertrophy [53].

In the present study, the participants were students. One of the limitations of the present study was the lack of (1) control over extracurricular activities outside the club and during school hours (e.g., potential but unlikely excessive physical activity during physical education lessons at school) and (2) sleep monitoring. Moreover, inflammatory factors influence the expression and amount of skeletal muscle growth-regulating factors [54]. The present study is part of a main project in which serum levels of interleukin-6 were measured, but we were not allowed to report them in this article. Therefore, we recommend that future researchers investigate inflammatory and proinflammatory factors simultaneously with measuring serum levels of myogenic and growth factors. On the other side, in addition to holding a briefing session for the participants and their parents to raise awareness of nutritional requirements, recovery, and research stages before the start of interventions and verbal questions and answers from the subject about nutrition and their sleep before each intervention session, there was the lack of nutritional, sleep and mood and anxiety control of participants. Also, because of our limitations in the number of EIA Kits and could include a maximum of 20 participants in the study, we ran the experiment with high quality and accuracy.

## Conclusion

Our results indicated that eight weeks of HICT significantly decreased the resting serum values of MSTN in male adolescent soccer players but did not change the resting serum values of MyoG. HICT did not affect MyoG and MSTN response after maximum aerobic test showing no effect on acute responses. In conclusion, HICT may be an appropriate training protocol for maintaining muscle mass in male adolescent soccer players.

## Data Availability

The current data in this study are available on request from the corresponding author.

## Abbreviations

MSTN: Myostatin
MyoG: MyoGenin
HICT: High-intensity circuit training
MyoD: Myoblast determination
ActRIIB: Activin receptor type IIB
TAK-1: TGF-β-activated kinase 1
MAPKK: Mitogen-activated protein kinase kinase
p38 MAPKs: P38 mitogen-activated protein kinases
Ras: Rat sarcoma virus
Raf: Rapidly accelerated fibrosarcoma
MEK1: Mitogen-activated protein kinase kinase-1
ERK1/2: Extracellular signal‐regulated kinases1/2
RPE: Rating of perceived exertion
EIA: ELISA
GASP-1: Growth and differentiation factor-associated serum protein-1
FOXo-3a: Forkhead transcription factor O subfamily member 3a
TG: Training group
CG: Control group
MRFs: Myogenic regulatory factors
TGF-B: Transforming growth factor-beta
GDF-8: Growth/differentiation factor-8
BMI: Body mass index
PAR-Q: Physical Activity Readiness Questionnaire
APHV: Age of peak height velocity
